# Pulmonary Rehabilitation: Time for an Upgrade

**DOI:** 10.3390/jcm9092742

**Published:** 2020-08-25

**Authors:** Raquel Sebio-García

**Affiliations:** 1 Department of Rehabilitation, Hospital Clínic de Barcelona, 08036 Barcelona, Spain; raquelsebio@gmail.com; 2 Grup de Recerca en Atenció a la Cronicitat i Innovació en Salut, School of Health Sciences TecnoCampus, University Pompeu Fabra, 08036 Barcelona, Spain

**Keywords:** pulmonary rehabilitation, COVID-19, tele-rehabilitation

## Abstract

Pulmonary rehabilitation is a notoriously known but highly underused intervention aimed to restore or improve functional capacity, symptom management and health-related quality of life among patients with chronic respiratory diseases. Since early 1980s, pulmonary rehabilitation has been acknowledged as a comprehensive intervention with hundreds of studies being performed over the past thirty years demonstrating its benefits on multiple outcomes; nevertheless, there are still multiple unresolved challenges, and new ones are currently emerging, with the COVID-19 outbreak now in the spotlight. In this editorial, these issues are summarized and discussed, while presenting some of the latest findings in research and clinical practice, with the ultimate goal of raising awareness of the future of pulmonary rehabilitation in the post COVID-19 era.

## 1. Introduction

Pulmonary rehabilitation (PR) is a well-established, widely known intervention that needs little introduction among the research community especially for those working with chronic respiratory diseases. PR stems from a comprehensive evaluation of the patient aimed to design an individually-tailored, multi-component intervention to optimise symptom control, pulmonary function, exercise capacity and health-related quality of life [1]. As a multi-component intervention, PR consists of different elements including but not limited to exercise training (both endurance and resistance), breathing exercises, smoking cessation, education as well as psychological and nutritional support, among others [2]. The frequency, intensity and method of delivery of these components might vary between individuals depending on their characteristics and specific needs but the ultimate focus should be always improving patient’s life and achieving behavioural change. 

The effectiveness of PR on different outcomes such as exercise capacity, muscle function, dyspnoea and symptom control, is quite robust, so it is currently recommended in the management of different chronic respiratory conditions, especially for patients with chronic obstructive pulmonary disease (COPD). In 2001, the Global Initiative for Obstructive Lung Disease (GOLD) endorsed PR as a standard of care for people with COPD. Furthermore, PR is the most cost-effective intervention along with smoking cessation for patients with COPD [3]. Currently, there is also evidence that PR might improve prognosis of the disease by reducing exacerbations, readmissions and potentially mortality [4,5]. However, despite the bulk of evidence showing the benefits of PR and its acknowledgement as a core therapy in patients with chronic respiratory diseases, there are still unresolved issues and knowledge gaps that need our attention. 

## 2. Current Challenges in Pulmonary Rehabilitation

One of the core difficulties that PR faces is its lack of and limited access to programmes for a large population of patients worldwide. In a study conducted in the US, only 2.7% of patients were referred to a pulmonary rehabilitation programme within 12 months of a COPD exacerbation [6]. Reasons for this underutilization are several but commonly identified issues include insufficient funds, lack of awareness/referral of patients, inadequate allocation of healthcare resources, lack of specialised healthcare professionals and/or adequate training opportunities [7]. In 2015 the American Thoracic Society and the European Respiratory Society developed a policy statement in which they addressed these limitations and provided different strategies to improve implementation of PR [7]. In addition to these policies which largely depend on governments and funding bodies, new research has been recently conducted to increase delivery and uptake of pulmonary rehabilitation. For instance, Marques and colleagues [8] have designed a real-world non-randomised controlled study, where they plan to engage primary healthcare centres where programmes are not available, by training healthcare professionals in the basics components of PR. This programme has the benefit of bringing PR closer to the community of the patients, thus reducing commuting times to hospital, and therefore improving both access and adherence. Home-based PR has also been studied as a feasible option to increase the delivery of PR especially among patients who live far from their hospital of reference, and/or have problems commuting because of their physical limitations, or cannot rely on their caregivers to travel. Several studies have shown that home-based PR can be as effective as supervised face-to-face sessions to improve exercise capacity and health-related quality of life in patients with COPD [9], while others have found no effects [10]. Choosing the most appropriate setting according to patients’ characteristics (stage of the disease, transportation options, stable or unstable, degree of disability) and goals (maintenance, improvement, etc.) is definitely an important gap in the current literature, which should be tackled to improve the uptake of PR. 

Another unanswered problematic that has been extensively investigated over the past years is how to maintain the benefits achieved during the programme in the long term. It is commonly acknowledged that if no maintenance strategy is provided, the benefits achieved during PR are likely to disappear after 6 to 12 months [1]. Among the potential ways to extend the benefits of PR the use of telemedicine and medical technology has gained quite popularity. In a recent study published by Jimenez et al. [11], the addition of a mHealth application with a patient-educator interaction following a pulmonary rehabilitation programme of 12 weeks resulted in an increase in adherence to respiratory physiotherapy treatment compared to a control group. Technology-supported exercise interventions, with the addition of a monitoring device, such as a pedometer or a fitness tracker, have shown to be effective in improving physical activity when a target is set (for example, 10.000 steps per day) [12]. However, the success of these devices in improving or maintaining physical activity and/or exercise capacity are likely to be subject to the support of a behavioural change intervention. Therefore, a multi-disciplinary team should be monitoring and supporting the use of this technology instead of just giving it to the patient and “let him be”.

## 3. Future Directions

Undoubtedly, the biggest challenge that PR is facing today and will continue to do so in the future is the COVID-19 outbreak, which has had a tremendous impact on the healthcare systems around the world and has dramatically affected not only those diagnosed by the disease but also those vulnerable or at higher risk. As of August 12th, more than 20 million people have been diagnosed with the disease, and more than 740.000 have died worldwide. People surviving the disease, especially those that have been admitted to the ICU, are at risk of developing long-term complications and sequelae, such as pulmonary fibrosis, persistent dyspnoea, impaired pulmonary function, decreased functional and exercise capacity, as well as neurological and cognitive impairments. According to a study conducted in Italy, at the time of being discharged from the hospital the one-minute sit-to-stand was below the 2.5th percentile of the reference values in 33% of the patients. In addition, the Barthel index which measures participation and limitation in activities of daily living (ADLs) was found to be poor in almost half the patients (64% were dependent for bathing, 24 for dressing/undressing, 35% for toilet use, 35% were immobile and 30% were wheelchair dependent, while 17.5% were still bedridden) [13]. As time goes by since the beginning of the pandemic, more studies are being published highlighting the long-term effects of the disease and the potential role of rehabilitation. In a recent cross-sectional study conducted among patients who had been hospitalised for COVID-19, more than half of patients were still experiencing fatigue, breathlessness, and decreased health-related quality of life 48 days after discharge [14]. This situation comes as no surprise as evidence from the 2003 SARS as well as ICU survivors show that these patients are at a high risk of developing post-intensive care syndrome (PICS) which is characterised by the presence of cognitive, psychiatric and physical impairments. There’s an urgent need to provide an adequate response to the demands that this population is facing with the on top difficulty of adapting traditional face-to-face rehabilitation to other delivery platforms as we continue under the social distancing premises. The COVID-19 should work as a “wake-up call” for governments and healthcare systems to implement telehealth solutions including tele-rehabilitation and remote monitoring of patients in preparation for future waves with potential new periods of isolation which will compromise again the ability to deliver PR interventions at the hospital or in the community. In addition to those who are already undertaking PR, rehabilitation services will also have to provide safe alternatives for the screening, inclusion and monitoring of new patients (both COVID-19 and non-COVID19) in this context. To this matter, experts on the field have recently proposed what kind of test should be used in a remote environment based on their safety and appropriateness. The short physical performance battery test (SPPB), the sit-to-stand test, the stair climbing test and the timed up and go test have thus been recommended both in those recovering from COVID-19 and also in other patients with respiratory conditions who are vulnerable and at risk [15]. Overall, there’s enough evidence to support the use of tele-rehabilitation as a safe, effective alternative to traditional PR not only as a short-term solution to the current situation but also to alleviate the burden on rehabilitation services and healthcare systems and increase reach to PR to more patients in need [16].

## 4. Conclusions

In summary, this is definitely an exciting year to contribute to the body of knowledge in pulmonary rehabilitation and embrace the challenges that it brings. We highly encourage researchers to submit your latest manuscript to this Special Issue in the ***Journal of Clinical Medicine***, a top open-access journal in its category and a reference for researchers in the different fields of medicine.

## References

[1] Spruit M.A., Singh S.J., Garvey C., ZuWallack R., Nici L., Rochester C., Hill K., Holland A.E., Lareau S.C., Man W.D.-C. (2013). An Official American Thoracic Society/European Respiratory Society Statement: Key Concepts and Advances in Pulmonary Rehabilitation. Am. J. Respir. Crit. Care Med..

[2] Troosters T., Demeyer H., Hornikx M., Camillo C.A., Janssens W. (2014). Pulmonary Rehabilitation. Clin. Chest Med..

[3] Zoumot Z., Jordan S., Hopkinson N.S. (2014). Emphysema: Time to say farewell to therapeutic nihilism. Thorax.

[4] Puhan M.A., Gimeno-Santos E., Cates C.J., Troosters T. (2016). Pulmonary rehabilitation following exacerbations of chronic obstructive pulmonary disease. Cochrane Database Syst. Rev..

[5] Ryrsø C.K., Godtfredsen N.S., Kofod L.M., Lavesen M., Mogensen L., Tobberup R., Farver-Vestergaard I., Callesen H.E., Tendal B., Lange P. (2018). Lower mortality after early supervised pulmonary rehabilitation following COPD-exacerbations: A systematic review and meta-analysis. BMC Pulm. Med..

[6] Spitzer K.A., Stefan M.S., Priya A., Pack Q.R., Pekow P.S., Lagu T., Pinto-Plata V.M., ZuWallack R.L., Lindenauer P.K. (2019). Participation in Pulmonary Rehabilitation after Hospitalization for Chronic Obstructive Pulmonary Disease among Medicare Beneficiaries. Ann. Am. Thorac. Soc..

[7] Rochester C.L., Vogiatzis I., Holland A.E., Lareau S.C., Marciniuk D.D., Puhan M.A., Spruit M.A., Masefield S., Casaburi R., Clini E. (2015). An Official American Thoracic Society/European Respiratory Society Policy Statement: Enhancing Implementation, Use, and Delivery of Pulmonary Rehabilitation. Am. J. Respir. Crit. Care Med..

[8] Marques A., Jácome C., Rebelo P.F.S., Paixão C., Oliveira A., Cruz J., Freitas C.M., Rua M., Loureiro H., Peguinho C. (2019). Improving access to community-based pulmonary rehabilitation: 3R protocol for real-world settings with cost-benefit analysis. BMC Public Health.

[9] Coats V., Maltais F., Simard S., Frechette E., Tremblay L., Ribeiro F., Saey D. (2013). Feasibility and Effectiveness of a Home-Based Exercise Training Program Before Lung Resection Surgery. Can. Respir. J..

[10] Lahham A., McDonald C.F., Moore R., Cox N.S., Rawlings S., Nichols A., Liacos A., Holland A.E. (2020). The impact of home-based pulmonary rehabilitation on people with mild chronic obstructive pulmonary disease: A randomised controlled trial. Clin. Respir. J..

[11] Jiménez-Reguera B., López E.M., Fitch S., Juarros-Monteagudo L., Sánchez-Cortés M., Rodríguez-Hermosa J.L., Calle-Rubio M., Hernández-Criado M.T., López-Martín M., Angulo S. (2020). Development and Preliminary Evaluation of the Effects of an mHealth Web-Based Platform (HappyAir) on Adherence to a Maintenance Program After Pulmonary Rehabilitation in Patients With Chronic Obstructive Pulmonary Disease: Randomized Controlled Trial (Preprint). JMIR mHealth uHealth.

[12] Bravata D.M., Smith-Spangler C., Sundaram V., Gienger A.L., Lin N., Lewis R., Stave C.D., Olkin I., Sirard J.R. (2007). Using Pedometers to Increase Physical Activity and Improve Health: A systematic review. J. Am. Med. Assoc..

[13] Belli S., Balbi B., Prince I., Cattaneo D., Masocco F., Zaccaria S., Bertalli L., Cattini F., Lomazzo A., Negro F.D. (2020). Low physical functioning and impaired performance of activities of daily life in COVID-19 patients who survived the hospitalisation. Eur. Respir. J..

[14] Halpin S.J., McIvor C., Whyatt G., Adams A., Harvey O., McLean L., Walshaw C., Kemp S., Corrado J., Singh R. (2020). Post-discharge symptoms and rehabilitation needs in survivors of COVID-19 infection: A cross-sectional evaluation. J. Med. Virol..

[15] Holland A.E., Malaguti C., Hoffman M., Lahham A., Burge A.T., Dowman L., May A.K., Bondarenko J., Graco M., Tikellis G. (2020). Home-based and remote exercise testing in chronic respiratory disease, during the COVID-19 pandemic and beyond: A rapid review. medRxiv.

[16] Jácome C., Marques A., Oliveira A., Rodrigues L., Sanches I. (2020). Pulmonary telerehabilitation: An international call for action. J. Clean Prod..

